# Correctional nursing in Canada’s Prairie provinces: Roles, responsibilities, and learning needs

**DOI:** 10.1177/0844562121999282

**Published:** 2021-03-03

**Authors:** Phil Woods, Cindy Peternelj-Taylor

**Affiliations:** 1 College of Nursing, University of Saskatchewan, Saskatoon, Saskatchewan, Canada

**Keywords:** Correctional nursing, learning needs assessment, nursing interventions, roles and responsibilities, incarcerated persons

## Abstract

**Background:**

Nurses represent the largest group of health care professionals working with incarcerated persons, yet there is limited understanding of their learning needs, or their roles and responsibilities; and what is known is poorly disseminated.

**Purpose:**

The goal of this research was to describe the roles, responsibilities, and learning needs of correctional nurses practicing in provincial correctional facilities in Alberta and Manitoba, and to add these data to the existing data set from Saskatchewan.

**Methods:**

Three hundred and forty nurses working in provincial correctional facilities in western Canada were invited to complete a self-administered online survey consisting of a Learning Needs Assessment questionnaire (demographic information, knowledge and learning needs, and professional development); and the Staff Questionnaire (which targeted specific skill sets relevant to clinical practice in secure environments). Eighty-two nurses completed the online survey (overall response rate 24%).

**Results:**

Overall, those who participated were experienced in nursing and correctional nursing. The learning needs they identified aligned with their correctional nursing roles and unique practice settings. In particular, issues related to the care of incarcerated persons with mental health disorders and related care were paramount (self-harming behaviours, suicide, mental health assessments in general). In response to the five comprehensive skill sets assessed in the Staff Questionnaire, respondents rated their involvement and importance of the individual skills as important to varying degrees.

**Conclusions:**

The results of this survey shed light on contemporary developments in correctional nursing within provincial correctional facilities in western Canada and provide a foundation for continuing professional education and development, practice, and future research initiatives.

## Introduction

The provision of correctional health care around the globe provides unique challenges for all professionals involved. This is partly owing to the nature of the correctional population. For instance, not only do incarcerated persons experience complex physical, mental health, and psychosocial needs and issues; the crimes they commit, the risks associated with these, and the formulation of plans of care to support them can be especially difficult. Nurses, whilst not the only profession to provide supportive care, they are perhaps the largest group. Within Canada, correctional nurses practice in two similar, yet distinct, correctional systems: federal and provincial/territorial corrections. The Correctional Service Canada, is the federal government agency responsible for the incarceration and rehabilitation of persons convicted of criminal offenses and sentenced to two years or more. Provincial and territorial corrections are generally responsible for persons who have been remanded in custody while awaiting trial, and those charged and sentenced by the courts to less than two years.

On a global scale, nurses have practiced in correctional environments since the late 1960s and early 1970s. However, as a workforce, their contributions have been largely hidden - publicly and professionally (Peternelj-Taylor, 2020). And although correctional nurses find themselves practicing in one of the most challenging environments for the profession, professional development within this specialty has been largely anecdotal, as early scholars often based professional development on expert opinion. However, research has been slowly emerging to grow the evidence-base. Examples from this research have focussed on professional role definition and challenges encountered within the context of care.

Flanagan and Flanagan (2001) examined American correctional nurses’ perceptions of their roles in relation to prisoner health care and training needs. Respondents to their survey reported that the most immediate and important training needs for correctional nurses were: continuous upgrading of assessment skills, techniques to interact with difficult patients, training on security related issues, and education on common health problems seen in the correctional setting. Shelton (2009) studied the role of correctional nurses (N = 180) from fourteen states across the United States. Participants’ responses to a self-administered questionnaire saw the emergence of two main roles: 1) the promotion and implementation of principles that underpin effective quality care for individual patients; and 2) the assessment, development, implementation and improvement of programs of care for individuals. In a Canadian study, Almost et al. (2013) found several work-life issues affected professional development for Ontario provincial correctional nurses: inadequate staffing and heavy workloads; limited control over their practice and scope of practice; limited resources, and challenging workplace relationships. More recently, Schoenly (2015) examined research priorities for correctional nursing practice. Through the application of a Delphi methodology, six priorities emerged: critical thinking and clinical judgment, competency and educational level, assessment, nursing protocols, effect on patient outcomes, and the environment of care.

We previously reported on a research study that addressed Saskatchewan provincial correctional nurses’ roles, responsibilities, and learning needs (Peternelj-Taylor & Woods, 2019). In this paper, we report on a follow-up study, an extension of this initial research, which included correctional nurses practicing in two other western provinces: Alberta and Manitoba.

## Goal and aims

As a continuation of our previous research, our goal was to describe the roles, responsibilities, and learning needs of correctional nurses practicing in provincial correctional facilities in Alberta and Manitoba, and to add these data to the existing data set from Saskatchewan. Specifically, we aimed to:

1. Describe the characteristics of correctional nurses in Canada’s prairie provinces; and
2. Identify their learning needs and the factors that influenced their ability to work to their full scope of practice.

## Method

 An email was sent out via the office of the nursing administrators in each province, to the population of nurses working in provincial correctional services in Manitoba (N = 95) and Alberta (N = 150) inviting them to complete an online Fluid Survey. The survey included two questionnaires: a Demographics & Learning Needs Assessment questionnaire (Peternelj-Taylor & Woods, 2019); and the Staff Questionnaire (United Kingdom Central Council & University of Central Lancashire [UKCC & UCL], 1999). Emails were sent out to all the nurses as a reminder to complete the survey.

Through the online recruitment process respondents were provided with details of the study and given the opportunity to ask questions. Free and implied informed consent was noted through completion and submission of the online Fluid Survey. Ethical approval was received through the Research Ethics Office at the University of Saskatchewan, Health Research Ethics Board of Alberta, and Manitoba Justice Community Safety Division.

### Sample

A total of 22 surveys were completed in Manitoba (response rate 23.2%) and 27 were completed in Alberta (response rate 18%). Responses (N = 33) from a similar previous survey of 95 Saskatchewan correctional nurses (response rate 34.7%) were also included (Peternelj-Taylor & Woods, 2019). A total of 82 participants made up the total sample of respondents from all three provinces used for analysis.

### Instrumentation

The Learning Needs Assessment developed by Faculty from the College of Nursing, University of Saskatchewan, was adapted for use in the online Fluid Survey. The survey included twenty-three questions: nine covering demographic information; eight covering knowledge and learning needs using a five-point Likert-type scale; five covering ongoing professional development; and one final open question seeking any additional comments. The Staff Questionnaire, developed for use in the Nursing in Secure Environments project (UKCC & UCL, 1999), includes 45 questions targeting five skill sets relevant to clinical practice: 1) promote and implement principles that underpin effectiveness, quality, and practice; 2) assess, develop, implement and improve programs of care for individuals; 3) create and maintain environments and relationships with individuals that value them as people and support their therapeutic roles; 4) provide and improve resources and services that facilitate organizational functioning; and 5) develop the knowledge, competency, and practice of self and other. For each of the 45 questions respondents rated their level of involvement within their professional role, and then rated using a Likert-type scale how important each skill set was to their role. As both are intended to be descriptive questionnaires evidence is not available for their validity and reliability.

### Data analysis

Data were cleaned and entered into the existing Saskatchewan data set that was previously reported by Peternelj-Taylor and Woods (2019). Data analysis was undertaken using IBM SPSS Statistics Version 26. For study aim one, analysis comprised of descriptive statistics (frequency, percentages, mean, standard deviation, and range). Group differences were compared using relevant parametric and non-parametric statistical tests: professional nursing designation, gender, full or part-time status (chi-square test); age and years of nursing experience (ANOVA). For study aim two, likewise analysis comprised of descriptive statistics (frequency, percentages, mean, standard deviation, and range). For the staff questionnaire group differences were compared for part of role versus not part of role (chi -square test or Fisher-Freeman-Halton exact test), and importance of staff questionnaire interventions between provinces (Kruskal-Wallis test, with Dunn’s pairwise test adjusted with Bonferroni correction for multiple tests) to determine where any significant differences. Statistical significance was set at p < 0.05.

## Results

### The characteristics of correctional nurses in the Canada’s Prairie provinces

The demographic responses are summarized in Table 1. Most respondents were Registered Nurses [RN], followed by Registered Psychiatric Nurses [RPN]. There was some variation between the three provinces, but none were significant. Not surprisingly there was a significantly higher number of female nurses who took part in the study as opposed to male respondents [77% versus 23%, *X*^2^ (2, N = 82) = 23.610, p < 0.001]. The mean age of the respondents was 41.35 years old. Manitoba respondents were significantly older [F(2, 79)=5.96, p = 0.004]. Overall, more respondents worked full-time as opposed to part-time [58% versus 42%]. While some provincial variation was evident, only in Manitoba did more respondents work part-time; however, the difference was only minimal. For over half of the respondents their highest level of education was at the degree level [55%]; while 39% of the respondents reported they were educated at the diploma level. There was a fair amount of variation in these two levels between the three provinces. Respondents had many years experience in nursing, and many years experience in correctional nursing. Of note, respondents from Manitoba had significantly more experience in nursing [F(2, 75)=3.97, p = 0.023]. At the time of survey completion, most respondents worked with an adult correctional population. Only a small number of respondents worked with a youth correctional population.

**Table 1.** Summary of demographics.

	Alberta	Manitoba	Saskatchewan	All
Professional designation				
RN	N = 20 (74.1%)	N = 10 (47.6%)	N = 15 (45.5%)	N = 45 (55.6%)
RPN		N = 8 (38.1%)	N = 14 (42.4%)	N = 22 (27.2%)
RN/RPN			N = 3 (9.1%)	N = 3 (3.7%)
LPN	N = 6 (22.2%)	N = 3 (14.3%)		N = 9 (11.1%)
Other	N = 1 (3.7%)		N = 1 (3.0%)	N = 2 (2.5%)
Gender				
Female	N = 23 (85.2%)	N = 14 (63.6%)	N = 26 (78.8%)	N = 63 (76.8%)
male	N = 4 (14.8%)	N = 8 (36.4%)	N = 7 (21.2%)	N = 19 (23.2%)
Age	Mean 38.59 (SD = 10.67, Min-=22, Max = 62)	Mean 48.00 (SD = 7.80, Min-=27, Max = 59)	Mean 39.18 (SD = 11.99, Min-=24, Max = 60)	Mean 41.35 (SD = 11.20, Min-=22, Max = 62)
Employment status				
Full-time	N = 16 (69.6%)	N = 14 (63.6%)	N = 15 (46.9%)	N = 45 (58.4%)
Part-time	N = 7 (30.4%)	N = 8 (36.4%)	N = 17 (53.1%)	N = 32 (41.6%)
Highest level of education				
Certificate	N = 1 (3.7%)	N = 4 (18.2%)		N = 5 (6.1%)
Diploma	N = 6 (22.2%)	N = 11 (50%)	N = 15 (45.5%)	N = 32 (39.0%)
Degree completed	N = 20 (74.1%)	N = 7 (31.8%)	N = 18 (54.5%)	N = 45 (54.9%)
Graduate degree in progress	N = 1 (3.7%)	N = 1 (4.5%)	N = 2 (6.1%)	N = 4 (4.9%)
Length of experience in nursing	mean 14.44 (SD = 11.69, Min = 1, Max = 42)	mean 21.63 (SD = 11.37, Min = 4, Max = 38)	mean 12.75 (SD = 12.04, Min = 0.5, Max = 40)	mean 15.75 (SD = 12.20, Min = 0.5, Max = 42)
Length of experience in correctional nursing	mean 7.65 (SD = 9.66, Min = 0.5, Max = 37)	mean 5.90 (SD = 5.61, Min = 0.75, Max = 25)	mean 8.65 (SD = 9.70, Min = 0.42, Max = 33)	mean 7.58 (SD = 8.71, Min = 0.42, Max = 37)
Current correctional population working with				
Adult	N = 27 (100%)	N = 19 (86.4%)	N = 29 (87.9%)	N = 75 (91.5%)
Youth		N = 3 (13.6%)	N = 4 (12.1%)	N = 7 (8.5%)

### Knowledge/learning needs

#### Correctional assessment

Respondents considered all areas of correctional assessment as either important or very important to their knowledge/learning needs (between 80% to 94%). Mental health and suicide assessment emerged as the most important, closely followed by self-harm and physical assessment (see Table 2). There was some variation in responses between the three provinces. Respondents from Manitoba had a more even split between the importance of correctional assessment (between important or very important), whereas respondents in Alberta and Saskatchewan responded more as very important.

**Table 2.** The importance of knowledge/learning needs.

Area	Not at all important N (%)	Somewhat important N (%)	Neutral N (%)	Important N (%)	Very important N (%)
Correctional assessment					
Risk assessment		5 (6.1)	3 (3.7)	23 (28.0)	51 (62.2)
Mental status assessment		3 (3.7)	2 (2.4)	16 (18.3)	52 (75.6)
Addictions assessment		4 (4.9)	8 (9.8)	28 (34.1)	42 (51.2)
Suicide assessment		2 (2.4)	3 (3.7)	9 (11.0)	68 (82.9)
Self-harm assessment*		2 (2.5)	4 (4.9)	14 (17.3)	61 (75.3)
Violence assessment		5 (6.1)	8 (9.8)	19 (23.2)	50 (61.0)
Physical assessment		3 (3.7)	4 (4.9)	18 (22.0)	57 (69.5)
Harm reduction assessment		6 (7.3)	10 (12.2)	35 (42.7)	31 (37.8)
Therapeutic interventions					
Group	10 (12.2)	11 (13.4)	32 (39.0)	20 (24.4)	9 (11.0)
Individual	2 (2.4)	2 (2.4)	8 (9.8)	25 (30.5)	45 (54.9)
Psychoeducation	3 (3.7)	4 (4.9)	16 (19.5)	33 (40.2)	26 (31.7)
Special populations					
Aboriginal offenders	2 (2.4)	2 (2.4)	7 (8.5)	21 (25.6)	50 (61.0)
Women offenders*	9 (11.7)	2 (2.6)	12 (15.6)	22 (28.6)	32 (41.6)
Youth offenders*	16 (20.8)	3 (3.9)	19 (24.7)	20 (26.0)	19 (24.7)
Elderly offenders*	4 (4.9)	5 (6.2)	13 (16.0)	34 (42.0)	25 (30.9)
Palliative offenders*	5 (6.4)	9 (11.5)	16 (20.5)	27 (34.6)	21 (26.9)
Mentally ill offenders	1 (1.2)	2 (2.4)	2 (2.4)	14 (17.1)	63 (76.8)
Offenders with addictions issues	1 (1.2)	2 (2.4)	5 (6.1)	25 (30.5)	49 (59.8)
Sex offenders	1 (1.2)	7 (8.5)	18 (22.0)	26 (31.7)	30 (36.6)
Personality disordered offenders	1 (1.2)	3 (3.7)	8 (9.8)	21 (25.6)	49 (59.8)
Disabled/Intellectually impaired offenders	1 (1.2)	4 (4.9)	5 (6.1)	32 (39.0)	40 (48.8)
Acquired brain injured offenders*	1 (1.2)	5 (6.2)	6 (7.4)	36 (44.4)	33 (40.7)
FASD	1 (1.2)	4 (4.9)	7 (8.5)	28 (34.1)	42 (51.2)
Offenders with bloodborne infectious diseases	3 (3.7)	3 (3.7)	9 (11.0)	22 (26.8)	45 (54.9)
Offenders with other infectious diseases*	3 (3.7)	2 (2.5)	8 (9.9)	26 (32.1)	42 (51.9)
Chronically physically ill offenders*	2 (2.5)	3 (3.7)	11 (13.6)	28 (34.6)	37 (45.7)
Offenders with concurrent disorders*	1 (1.2)	3 (3.7)	10 (12.3)	27 (33.3)	40 (49.4)
Roles & Specialties					
Professional role development*	2 (2.5)		5 (6.3)	29 (36.3)	44 (55.0)
Career opportunities*	2 (2.5)	6 (7.5)	16 (20.0)	30 (37.5)	26 (32.5)
Roles of the interdisciplinary team*	2 (2.5)	5 (6.3)	4 (5.0)	27 (33.8)	42 (52.5)
Research and development*	3 (3.8)	5 (6.3)	13 (16.5)	40 (50.6)	18 (22.8)
Correctional or other relevant education available*	2 (2.6)	3 (3.8)	7 (9.0)	22 (28.2)	44 (56.4)
Practice Issues					
The therapeutic relationship*	2 (2.5)	4 (5.0)	10 (12.5)	26 (32.5)	38 (47.5)
Ethical/moral issues in correctional care*	1 (1.3)	2 (2.5)	4 (5.0)	23 (28.7)	50 (62.5)
Care and custody*	1 (1.3)	2 (2.5)	7 (8.8)	29 (36.3)	41 (51.2)
Restraint & segregation*	1 (1.3)	3 (3.8)	12 (15.0)	35 (43.8)	29 (36.3)
Trauma/emergency response*	1 (1.3)		4 (5.0)	17 (21.3)	58 (72.5)
Medication management*	1 (1.3)	1 (1.3)	5 (6.3)	21 (26.3)	52 (65.0)
Human rights/offender rights/victim rights*	1 (1.3)	2 (2.5)	13 (16.3)	31 (38.8)	33 (41.3)
Infectious disease management*	1 (1.3)	2 (2.5)	6 (7.5)	18 (22.5)	53 (66.3)
Documentation*	1 (1.3)		4 (5.0)	11 (13.8)	64 (80.0)
Information sharing*	1 (1.3)	1 (1.3)	8 (10.0)	19 (23.8)	51 (63.7)

*Some missing data (valid percent used).

Previously we reported that Saskatchewan respondents identified additional areas of assessment as: gang affiliation activity assessment; past medical or psychiatric history; and prenatal care and status, diabetic status, and parental custodial concerns (Peternelj-Taylor & Woods, 2019). Additional areas of assessment identified by the respondents from Alberta and Manitoba included: Advanced Cardiac Life Support; environmental/setting assessment; emergency medical/trauma assessment and care; critical thinking; and being able to accurately assess and make an accurate diagnosis and then to be able to prescribe the appropriate treatment following standing orders or in consultation with the physician.

#### Therapeutic interventions

Respondents placed more importance on individual and psychoeducation interventions for their knowledge/learning needs, rating them as important or very important; 85% and 72% respectively (see Table 2). Less than half of the respondents (44%) considered group interventions as important or very important. There was some variation in responses between the three provinces, with Manitoba more neutral towards group and psychoeducation interventions. Likewise, many respondents from Alberta and Saskatchewan were neutral towards group interventions. One additional area of knowledge was identified: the importance of educating correctional operations regarding nursing interventions.

#### Special populations

Most of the respondents considered, knowledge/learning needs for fifteen of the special populations as either important or very important (between 62% to 94%). Only 51% of respondents considered the need for knowledge/learning need in the care of youth offenders as either important or very important. Knowledge around mentally ill offenders and offenders with addictions issues was rated by over 90% of respondents as either important or very important. Over 85% of respondents rated knowledge around disabled/intellectually impaired incarcerated persons, those who were Indigenous, those with personality disorder, Fetal Alcohol Spectrum Disorder (FASD), and persons with acquired brain injuries similarly (see Table 2). A small percentage of respondents (21%) considered knowledge around criminal justice involved youth to be not at all important. These respondents mainly did not work with a youth population.

There was variation within and between all three provinces as to how many respondents felt the knowledge/learning need was either important or very important for the identified special populations. Respondents from Manitoba were more inclined to rate knowledge around women, youth, and palliative offenders as neutral, or not at all important. Previously we reported that Saskatchewan respondents who participated in the study identified Post-traumatic Stress Disorder (PTSD) and spousal abuse as topic areas where additional knowledge was needed (Peternelj-Taylor & Woods, 2019). No additional special populations were identified by the Alberta and Manitoba respondents.

#### Role and specialities

Respondents considered professional role development, career opportunities, roles of the interdisciplinary team, research and development, and correctional or other relevant education available as either important or very important for knowledge/learning need of correctional nurses (see Table 2). While some provincial variation existed between the level of importance in these five areas, respondents from Manitoba tended to answer more in the neutral category.

Previously we reported that Saskatchewan respondents identified additional roles and specialities where knowledge is important: continued education and community awareness, importance of contextual issues outside of the facility, and the role of the nursing manager (Peternelj-Taylor & Woods, 2019). Additional knowledge on roles and specialities identified as important by the respondents from Alberta and Manitoba were: “collaborative practice”; and “working with the officers and other corrections staff as they often have different priorities than nursing and it is not always easy to work in collaboration”.

#### Practice issues

Ninety percent of the respondents considered knowledge of documentation, trauma/emergency response, medication management, and ethical/moral issues in correctional care to be either important or very important. For the remaining six practice areas of infectious disease management, information sharing, care and custody, human rights/offender rights/victim rights, restraint and segregation, and the therapeutic relationship, over eighty percent rated these the same (see Table 2). Provincial variation existed between the level of importance for most of these practice areas. For all the practice areas there were many neutral responses from the participants from Manitoba. In Alberta, there were also many neutral responses for the therapeutic relationship, restraint and segregation, and human rights/offender rights/victim rights.

Respondents identified topics where additional knowledge would be relevant to their work. In Saskatchewan, the respondents identified a number of specific topics they deemed significant to their role including: prenatal management; team development, critical incident support and debriefing, PTSD; diabetes management; effects of shift work, and work-life balance (Peternelj-Taylor & Woods, 2019). A few additional comments were made by the respondents from Alberta and Manitoba. A couple in particular stood out: “working with other corrections staff is sometimes a challenge due to differing priorities and that, often, the role of nursing is not understood”; and “we are sometimes considered glorified waitresses”.

#### In-service education and development

Just over 52% of respondents reported that their facility offered in-service education. There was some variation between provinces: Saskatchewan 42% (Peternelj-Taylor & Woods, 2019), Manitoba 45%, and Alberta 72%. Saskatchewan respondents identified resources available in the workplace for professional development as somewhat limited and according to one of our participants “NONE! It’s the responsibility of staff to seek out educational opportunities and then go through the ‘red tape’ of trying to get funding and the time off” (Peternelj-Taylor & Woods, 2019, p.184). Another respondent indicated that “Health care is not a priority for the provincial service in my opinion” and “Very limited due to supposed “budget issues” - little to no appreciation for professional development”.

We received similar responses from the respondents from the two other provinces. From Manitoba, one respondent noted: “In reality, not many. Certain courses have been offered but due to staffing requirements, I have not been allowed the time to take any of the courses which have been offered. We are severely short staffed”. While another shared that “The only resources made available are the ones that a few of the nurses brought in or developed. We bring in speakers when we can, but they must be free”. Another respondent simply acknowledged: “Little is suggested, promoted or encouraged”.

The comments from the Alberta respondents at first glance seemed more encouraging as stated by one respondent who noted that there was a: “Clinical Nurse Educator (CNE) onsite, use of Professional Development and education days off to attend conferences, seminars etc., access to the vast Alberta Health Services education services and references online and in person”. Another stated that they had “Ongoing in-services related to mental health, annual medical/mental health education, simulation training, [and] opportunity for involvement on site committees. We also have 2.5 CNEs that follow up with any staff requiring or requesting additional education”. Conversely, even though the Alberta correctional nurses had access to a clinical nurse educator, one of the respondents commented that education was: “Minimal. Our clinical nurse educator deals mostly with mandatory training and makes us aware of a few relevant opportunities”.

Overall, forty-four percent of respondents were aware of correctional nursing conferences that are offered. Provincial variation was large with Saskatchewan 58% (Peternelj-Taylor & Woods, 2019), Manitoba 25%, and Alberta 40%. Less than a quarter of respondents indicated that they had ever attended a conference on correctional nursing (23%). There was a fair amount of variation between provinces with Saskatchewan 33% (Peternelj-Taylor & Woods, 2019), Manitoba 10%, and Alberta 20%. Most respondents (96%) indicated that they would like to attend a conference on correctional nursing in the future (Saskatchewan 100%; Manitoba 85% and Alberta 100%).

#### Additional comments

Three themes were seen to emerge from the additional comments received from the respondents:

##### Saskatchewan emergent theme—Do more with less

- “The expectations of nurses increase but the number of nurse positions is decreased”.
- “Like most work places, nursing in corrections seems to have little or no funds to foster opportunities for staff development, even staff meetings seem difficult to arrange. It’s the old adage of doing more with less”.

##### Manitoba emergent theme—Desire for more education and collaboration

- “How nice and needed it would be to have a weekend conference directed at correctional nurses - one that would cover topics specific to our unique roles and one that would allow collaboration between the nurses from other correctional sites”.
- “Have been told that there is not budget for any education for health services staff. If we as individual nurses can find someone to come in and offer free education that is okay but don't expect anything else. There is a small pocket of provincial money, but it is very hard to get approval for this money and it is very limited in amount. The manager does nothing to help staff obtain funding to advance education”.
- “I didn't get correctional training. Bare essentials, not even what was or wasn't expected of me working in a correctional setting”.

##### Alberta—Emergent theme—Professional recognition

- “Correctional nursing is such a specialty that I am very surprised that there is not more offered in regard to professional development”.
- “The certification process in correctional nursing in the US brings a lot of prestige to the specialty; it would be great to see something like that in Canada”.
- “An acceptance of Corrections Nursing is long overdue in terms of the specialization required to work in this environment; others need to understand it is more than just handing out Motrin or Tylenol”.

### Scope of practice

As in the study by Peternelj-Taylor and Woods (2019) respondents rated 45 nursing interventions, which are grouped into five skills sets, for the the level of involvement they had within their work role, as well as how important they are for nursing in secure environments. Responses for level of involvement are: (1) part of role and ensure others undertake this; (2) ensuring others undertake this; (3) part of role; and (4) not part of role. Responses for how important the intervention is are measured on a Likert scale: very unimportant, unimportant, undecided, important, very important. As in our previous study (Peternelj-Taylor & Woods, 2019), a subset of results is reported, specifically those that reported the skill set intervention was “part of role & ensure others undertake this” or “part of role”; for simplicity, we refer to “part of role” from this point forward. In Table 3: “the percentage this represents of the total respondents for each item and the mean score and related statistics on how important this subset of respondents rated each item. Higher mean scores relate to higher importance (range 1–5)” (p. 184). Additionally, significant differences between provinces are reported in relation to the importance of each item for correctional nursing.

**Table 3.** Combined responses “part of role & ensure others undertake this” and “part of role.”

Skill set	N	% total item response	$\bar{x}$ Importance(Range 1-5)	SD	Provincial difference importance (Kruskal Wallis Test)
**Skill set 1. Promote and implement principles which underpin effective quality and practice**					
1: Promote people's equality, diversity and rights.	65	87.84	4.22	0.95	H(2)=6.95,p = 0.03
2: Promote effective communication and relationships.	62	86.11	4.16	1.33	H(2)=0.87,p = 0.65
3: Promote communication with individuals where there are communication differences.	58	80.56	4.21	0.96	H(2)=0.41,p = 0.81
**Skill set 2. Assess, develop, implement and improve programs of care for individuals**					
4: Assess individuals to determine their overall needs and risk.	66	91.67	4.17	1.29	H(2)=0.93,p = 0.63
5: Provide specialist assessment services on individuals' needs so that others can take action.	55	77.46	4.13	1.12	H(2)=2.69,p = 0.26
6: Assist in the assessment of and the planning of programs of care for individuals.	51	70.83	4.35	0.98	H(2)=5.17,p = 0.08
7: Plan specific therapeutic interventions to enable individuals to recognize and address any socially-unacceptable behaviour.	49	68.06	4.16	1.03	H(2)=2.70,p = 0.26
8: Contribute to the joint implementation and monitoring of programs of care for individuals.	46	67.65	4.17	0.85	H(2)=2.71,p = 0.26
9: Implement specific therapeutic interventions to enable individuals to manage their behaviour.	47	69.12	4.28	0.80	H(2)=0.49,p = 0.78
10: Assist in the implementation and monitoring of specific therapeutic interventions.	57	85.07	4.18	1.08	H(2)=1.58,p = 0.45
11: Enable individuals to develop and maintain skills of independent living.	42	63.64	4.24	0.86	H(2)=1.69,p = 0.43
12: Enable individuals to develop meaningful relationships with others.	28	43.75	4.04	0.88	H(2)=9.51,p = 0.01
13: Enable individuals who are at risk to themselves and others to identify behavioural boundaries and develop control.	50	73.53	4.20	0.93	H(2)=3.54,p = 0.17
14: Contribute to the evaluation and improvement of programs of care for individuals.	42	62.69	4.10	0.80	H(2)=0.60,p = 0.74
15: Assess individuals' needs for primary health care services.	63	91.30	4.40	1.04	H(2)=3.85,p = 0.15
16: Develop, monitor and review programs of primary health care for individuals.	45	65.22	4.20	1.05	H(2)=5.72,p = 0.06
17: Contribute to raising awareness of health issues.	65	94.20	4.25	0.94	H(2)=3.04,p = 0.22
18: Enable individuals to address issues which affect their health and well-being.	57	87.69	4.09	1.11	H(2)=5.31,p = 0.07
19: Raise awareness of the needs of individuals discharged from your services.	42	65.63	4.31	0.75	H(2)=1.44,p = 0.49
20: Promote the needs of individuals in the community.	30	47.62	4.27	0.87	H(2)=0.71,p = 0.70
21: Negotiate, agree and support placements for individuals.	31	47.69	4.09	1.15	H(2)=1.23,p = 0.54
22: Develop, monitor and review discharge packages to manage individuals.	28	43.08	4.11	0.92	H(2)=2.74,p = 0.25
**Skill set 3. Create and maintain environments and relationships with individuals which value them as people and support their therapeutic goals**					
23: Contribute to the provision of effective physical, social and emotional environments for group care.	29	48.33	3.86	1.16	H(2)=1.29, p = 0.53
24: Build and sustain relationships with individuals to reinforce their therapeutic goals.	50	78.13	4.12	0.77	H(2)=4.68,p = 0.10
25: Physically intervene in situations where there is a breakdown in environments and relationships to limit risks to those involved.	27	43.55	4.04	1.02	H(2)=4.04,p = 0.13
26: Support individuals with difficult or potentially difficult relationships.	40	62.50	4.00	0.88	H(2)=2.86,p = 0.24
27: Enable individuals to maintain contacts in isolating situations.	30	48.39	3.67	1.42	H(2)=3.04,p = 0.22
28: Enable individuals to adjust to and manage their loss.	47	75.81	3.91	1.18	H(2)=5.47,p = 0.07
29: Enable individuals’ partners, relatives and friends to adjust to and manage the individual’s loss.	12	19.67	3.67	1.23	H(2)=3.58,p = 0.17
30: Enable individuals, their partners, relatives and friends to explore and manage change.	15	24.59	3.71	1.38	H(2)=3.11,p = 0.21
31: Contribute to establishing and running mutual support networks.	18	31.03	3.76	1.30	H(2)=1.58,p = 0.45
32: Support individuals when they are distressed.	56	90.32	4.24	1.14	H(2)=2.47,p = 0.29
33: Create and maintain boundaries between the community and individuals detained in secure conditions.	32	56.14	4.16	1.10	H(2)=4.20,p = 0.12
34: Protect patients from themselves and each other.	50	80.65	4.38	1.05	H(2)=4.05,p = 0.13
35: Contribute to the protection of individuals from abuse.	43	69.35	4.33	1.02	H(2)=0.84,p = 0.66
36: Escort patients within and beyond secure settings.	7	11.48	4.00	1.41	Unable to compute
Skill set 4. Provide and improve resources and services which facilitate organizational functioning					
37: Manage one’s caseload against the prioritized needs of individuals.	37	66.07	4.19	1.10	H(2)=1.69,p = 0.43
38: Support and lead teams to enable work objectives to be met.	37	64.91	4.08	1.01	H(2)=4.55,p = 0.10
39: Support staff in maintaining their identity and safe personal boundaries.	39	67.24	4.31	0.95	H(2)=1.64,p = 0.44
40: Counsel and support staff in times of stress.	36	62.07	4.22	1.05	H(2)=7.31,p = 0.03
41: Promote, monitor and maintain health, safety and security in the workplace.	50	84.75	4.47	0.98	H(2)=2.67,p = 0.26
42: Receive, transmit, store and retrieve information.	54	93.10	4.31	1.01	H(2)=0.68,p = 0.71
**Skill set 5. Develop the knowledge, competence and practice of self and others**					
43: Contribute to the development of knowledge and practice.	51	86.44	4.36	1.05	H(2)=1.44,p = 0.49
44: Develop oneself within the role.	53	89.83	4.45	0.85	H(2)=3.91,p = 0.14
45: Contribute to the development of others.	43	75.44	4.21	0.86	H(2)=3.30,p = 0.19

As can be seen from the percentage total item response in Table 3 for many interventions in the five skill sets these are part of the respondent roles (14 over 80%, 6 over 70%, 13 over 60%). For the remaining interventions, for eight of these it was part of their role for between 40 to 50 percent of the respondents. However, for the remaining four interventions few respondents reported they were part of their role (interventions 29, 30, 31 and 36). These were all part of the third skill set “create and maintain environments and relationships with individuals which value them as people and support their therapeutic goals”.

While there was some variation between provinces in relation to the interventions being part of their roles, only a few were significant. These were:

- item 2 “promote effective communication and relationships” (Saskatchewan 96.88%, Manitoba 73.68%, Alberta.80.95%) [Fisher-Freeman-Halton exact test p=0.03, two-tailed].
- item 13 “enable individuals who are at risk to themselves and others to identify behavioural boundaries and develop control” (Saskatchewan 93.55%, Manitoba 63.16%, Alberta 50%) [*X*^2^ (2, N=68) = 12.553, p=0.002].
- item 19 “raise awareness of the needs of individuals discharged from your services” (Saskatchewan 82.14%, Manitoba 44.44%, Alberta 61.11%) [*X*^2^ (2, N=64) = 7.129, p=0.028].
- item 20 “promote the needs of individuals in the community” (Saskatchewan 70.37%, Manitoba 27.78%, Alberta 33.33%) [*X*^2^ (2, N=63) = 9.917, p=0.007].
- item 21 “Negotiate, agree and support placements for individuals” (Saskatchewan 64.29%, Manitoba 26.32%, Alberta 44.44%) [*X*^2^ (2, N=65) = 6.647, p=0.036].
- item 34 “protect patients from themselves and each other” (Saskatchewan 89.29%, Manitoba 61.11%, Alberta 87.5%) [Fisher-Freeman-Halton exact test p=0.06, two-tailed].
- item 36 “escort patients within and beyond secure settings” (Saskatchewan 25%, Manitoba 0%, Alberta 0%) [Fisher-Freeman-Halton exact test p=0.01, two-tailed].
- item 41 “promote, monitor and maintain health, safety and security in the workplace” (Saskatchewan 92.86%, Manitoba 62.5%, Alberta 93.33%) [Fisher-Freeman-Halton exact test p=0.01, two-tailed].

Respondents from the three provinces felt that all the interventions in the five skills sets were important for nursing in secure environments as can be seen from the mean scores in Table 3. While there was some variation in the mean scores between the three provinces these were significant (p < 0.05) for three interventions. These significant differences were following a Dunn’s pairwise test:

- item one “promote people's equality, diversity and rights” (p=0.03, adjusted with Bonferroni correction) between Manitoba and Saskatchewan respondents but not between the other two paired respondent groups.
- item twelve “enable individuals to develop meaningful relationships with others” (p=0.01, adjusted with Bonferroni correction) between Manitoba and Saskatchewan respondents but not between the other two paired respondent groups.
- item forty “counsel and support staff in times of stress” (p=0.03, adjusted with Bonferroni correction) between Manitoba and Alberta respondents but not between the other two paired respondent groups.

## Discussion

To date, correctional nursing has attracted very few researchers, even though correctional environments, and their inhabitants, represent fertile ground for further development regarding the care of incarcerated persons, and the promotion of this specialty area of practice. This study, the first of its kind in Western Canada, provided contemporary data regarding the important roles and responsibilities that correctional nurses adopt in the provision of health care to incarcerated persons serving time within provincial correctional settings. Correctional nurses are the backbone of correctional healthcare, which further underscores the importance of this study, with particular emphasis on their roles, responsibilities and learning needs. This study sheds light on the complexities inherent in the practice of correctional nursing from a vocational, educational, and professional practice perspective, and is foundational to the provision of competent evidence-informed care.

In particular, in reviewing the responses to the Learning Needs Assessment questionnaire (and combining both the important and very important responses), it was very clear how important knowledge is related to incarcerated persons with mental illness (94%), suicide assessment (94%), mental status assessment (94%), and self-harm assessment (93%). Similarly, it was also clear how important knowledge of the following special populations was: offenders with addiction issues (91%), Indigenous populations (87%), and personality disordered offenders (85%).

These findings in many ways are not surprising given the universal complexity of mental health and addiction issues experienced by incarcerated persons globally, and the challenges of providing appropriate care for this group within the correctional milieu. Moreover, these findings are consistent with other research and scholarship (Durcan & Zwemstra, 2014; Ellis & Alexander, 2017; Kolodziejczak & Sinclair, 2018; Kucirka & Ramirez, 2019; Reingle Gonzalez & Connell, 2014; Shelton et al., 2020). In Canada, as in other countries, criminalization of mental illness is a terse reality (Mental Health Commission of Canada, 2012; Peternelj-Taylor, 2008), where correctional facilities have become “defacto psychiatric institutions” (Chaimowitz, 2012, p. 5). Furthermore, incarcerated persons in Canada experience a higher burden of chronic illness, including mental illness, as well as traumatic brain injuries, and adverse events often experienced in early childhood (Kouyoumdjian et al., 2016). And while correctional nurse leaders are beginning to make headway in the care of incarcerated persons through the ongoing professional development of correctional nurses (Almost et al., 2020; Butler & Mallet-Boucher, 2020; Loeb et al., 2017; Simon et al., 2020), and the care of incarcerated persons with mental illness in particular (Ellis & Alexander, 2017; Maruca & Shelton, 2016; Mollard & Hudson, 2016; Shelton et al., 2017), the need for ongoing professional development and research in this niche area of practice remains a contemporary professional issue.

Historically, Indigenous persons have been disproportionately represented in Canadian correctional systems including provincial, territorial, and federal systems (Department of Justice, 2019; Peternelj-Taylor & Woods, 2020) the nurses who participated in our study, considered knowledge of Indigenous populations as either important or very important to their practice. As noted by the Department of Justice (2019), the “problem of overrepresentation of Indigenous adults in corrections is a general problem in most jurisdictions, particularly for remand and sentenced custody, the problem is more pronounced in the Western provinces” (⁋¶3). This underscores the importance of nurses in Alberta, Saskatchewan, and Manitoba, embracing cultural safety in their work as correctional nurses with Indigenous populations (Curtis et al., 2019; National Aboriginal Health Organization, 2006).

Responses to the Staff Questionnaire, for the most part, showed that for all five skills sets, the interventions were either part of their role, or part of their role and they ensured others undertook this. Where the percentages were lower, this could be due to the fact that traditionally these skill sets would not be considered something that correctional nurses might be expected to do as part of their role. There was little significant provincial variation. Likewise, the interventions in the five skill sets were considered important for correctional nursing practice, which is similar to other studies (Shelton, 2009; Shelton et al., 2010). Once again there was little significant provincial variation in responses.

The professional identity of nurses within this specialty domain warrants further discussion. In a guest editorial dedicated to nursing in the correctional milieu, Peternelj-Taylor (2020) concluded that “as a workforce they remain largely hidden and their professional contributions concealed behind prison walls, invisible to the public and the larger nursing community” (p, 1). It was interesting to note the comments regarding educational opportunities from the nurses who participated from Alberta, as well as their concerns re: their professional identity. Correctional nurses in provincial correctional facilities in Alberta, are employed by Alberta Health Services, unlike Saskatchewan and Manitoba, where correctional health care falls under the Ministry of Justice for Corrections and Policing, and Manitoba Justice respectively. The need to further raise the profile of correctional nursing was inherent in their commentary. Collaborative partnerships between academia and corrections are required to further enhance the profile of the correctional nurse, more fully address professional practice issues, and further contribute to the professional identity of the corrections nurse, thereby attracting “fresh blood” (Goddard et al., 2019, p. 168) into the specialty.

The research reported herein is the first of its kind in Western Canada and as such is important in understanding the challenges correctional nurses face in their day to day practice in the provision of nursing care to incarcerated persons in provincial correctional facilities. The exploration of correctional nurses learning needs and professional role development within the context of their unique work environments provides foundational information that will direct future practice developments, research, and innovations in workforce development and continuing education, and contribute to evidence informed quality care for incarcerated persons within Western Canada. The ongoing evolution of correctional nursing as a specialty is dependent on nursing leaders to promote and nurture the development of a nursing culture that embraces nursing research; facilitates the translation and interpretation of research relevant to practice within the correctional milieu; and engage in the collaborative identification of research questions for further study (Dhaliwal & Hirst, 2016; Peternelj-Taylor, 2020; Peternelj-Taylor & Woods, 2020; Shelton et al., 2020).

Unlike correctional nurses in the United States, whose practice is guided by the American Nurses Association’s (2020) *Correctional Nursing: Scope and Standards of Practice* (now in its third edition), and their ability to become certified by the National Commission of Correctional Health Care (see https://www.ncchc.org/CCHP-RN), first as a Certified Correctional Health Professional (CCHP), and then as a CCHP-RN, comparable standards or certification processes are nonexistent in Canada. However, the Ontario Correctional Nurses’ Interest Group, a member of the Registered Nurses’ Association of Ontario, contributes to the professional recognition of correctional nurses in Ontario, through “[fostering] knowledge-based correctional nursing practice, [promoting] quality work environments, [supporting] professional development of correctional nurses, and [advancing] healthy public policy” (see https://chapters-igs.rnao.ca/interestgroup/35/about). Regrettably, similar special interest groups are nonexistent in Alberta, Saskatchewan, and Manitoba.

As noted in their responses to our open-ended survey questions, the correctional nurses in this study longed for opportunities to enhance their professional practice through continued education and professional development; they also poignantly identified the many barriers that prevented them from moving in this direction. While nurses spoke of their desires to attend relevant professional conferences, the stark realities of their workplace budgets, and staff shortages, made conference attendance impossible to achieve. Increasingly nursing leaders, are exploring other venues for continuing education, including online educational interventions (Almost et al., 2019; Kitt-Lewis et al., 2020) and simulation-based education (Diaz et al., 2019).

Survey studies such as this, are conducted knowing that there are inherent limitations. The response rate while considered acceptable for surveys, actually represented a small number of nurses, who participated from the three Prairie Provinces. And while we recognize that our findings are not generalizable to other correctional jurisdictions, we believe our results will resonate with correctional nurses, and assist others in exploring the issues surrounding roles, responsibilities, and learning needs specific to the practice of correctional nursing in other provincial and federal correctional jurisdictions. The Staff Questionnaire (UKCC & UCL, 1999), while used in previous research studies (Shelton, 2003, 2009), presented as a limitation in our survey. Given the length (45 questions) and complexity, feedback from participants indicted it was challenging to complete in a timely way in an online environment.

## Conclusion

Historically, nurses who chose to work in correctional environments often experienced professional stigma, as they were seen as second class nurses, unable to secure employment elsewhere (Peternelj-Taylor & Johnson, 1995). And while correctional nursing, as a specialized area of nursing practice, has experienced transformational developments in recent years, Shelton et al. (2020), in a review of nursing in the American Justice System, acknowledge professional and public stigma remains an ongoing obstacle to the future of correctional nursing. In this study, we focused on a niche, yet underserved area of nursing practice. The results of this survey shed light on contemporary developments in correctional nursing within provincial correctional facilities in western Canada and provide a foundation for continuing professional education and development, practice, and future research initiatives.
